# Utilizing the CFIR to Evaluate Rural COVID-19 Vaccine Uptake: Extension Personnel Perspectives

**DOI:** 10.3390/vaccines14090776

**Published:** 2026-09-05

**Authors:** Megan Undeberg, Ghazal Meratnia, Skylar Zelenko, Kimberly McKeirnan

**Affiliations:** College of Pharmacy and Pharmaceutical Sciences, Washington State University, Spokane, WA 99202, USA; gmeratnia@gmail.com (G.M.); skylar.zelenko@wsu.edu (S.Z.); kimberly.mckeirnan@wsu.edu (K.M.)

**Keywords:** COVID-19, vaccination uptake, rural health, university extension, Consolidated Framework for Implementation Research (CFIR)

## Abstract

**Background/Objectives:** Rural populations in the United States experienced consistently lower COVID-19 vaccination rates than urban populations during the pandemic. While barriers to vaccination have been well documented, less is known about facilitators of uptake in rural settings. This study aimed to identify facilitators of COVID-19 vaccination in rural communities from the perspectives of Washington State University (WSU) Extension personnel and to examine these factors using the Consolidated Framework for Implementation Research (CFIR) Outer Setting domain. **Methods:** A qualitative descriptive study was conducted using semi-structured key informant interviews with WSU Extension personnel. Twenty-one participants representing 34 of 40 Extension offices across Washington State were interviewed in Fall 2023. Interviews were transcribed, de-identified, and analyzed using deductive thematic analysis. Findings were organized into the seven CFIR Outer Setting constructs: critical incidents, local attitudes, local conditions, partnerships and connections, policies and laws, financing, and external pressures. **Results:** Perceived facilitators of COVID-19 vaccination were identified across all CFIR Outer Setting constructs. Trust emerged as a central theme, with participants reporting that vaccine acceptance was higher when information was delivered by trusted local sources, including Extension personnel, healthcare providers, community leaders, and informal networks. Participants described leveraging partnerships with public health agencies, schools, faith-based organizations, and community groups as enhancing outreach and information dissemination. Additional facilitators identified by participants included no-cost vaccine and personal protective equipment availability, accessible community-based vaccination sites, culturally appropriate educational materials, and coordinated communication systems. **Conclusions:** Rural vaccination efforts were supported by trust, strong community partnerships, and locally tailored strategies. These findings may inform future public health emergency responses in rural communities.

## 1. Introduction

In the United States, approximately one in five individuals lives in a rural area [1,2,3,4,5]. The federal government identifies at-risk communities using the Index of Medical Underservice (IMU), which generates a composite score based on provider-to-population ratios, poverty levels, the proportion of individuals ages 65 and older, and infant health indicators. The score ranges from 0 to 100 and helps determine the level of federal support allocated to a given area [6]. Areas classified as Medically Underserved Areas and Populations (MUA/P) are further used to characterize levels of need, particularly in rural regions. Medically Underserved Areas (MUAs) are broader geographic designations, typically applied to counties or census tracts with IMU scores of 62.0 or lower [7,8]. In contrast, Medically Underserved Populations (MUPs) refer to specific groups that face barriers to accessing care regardless of geographic locations [7,8].

Rurality is a challenging label to apply, and it is dependent on many factors. In the United States the USDA states rural America are those areas that are not urban. To provide context, rural towns are “places with fewer than 5000 people and 2000 housing units” [9]. Rural communities across the United States experience substantial disparities in access to healthcare services [10]. Notably, approximately 80% of rural residents are considered medically underserved [11]. Even for preventive services such as vaccinations, individuals living in rural areas are less likely to receive routine immunizations than those in urban areas [12]. National data indicate that during the first year of COVID-19 vaccine availability, 58.5% of rural residents were vaccinated, compared to 75.4% among urban residents [13]. Similarly, a mid-year study reported vaccination rates of 45.8% among rural populations versus 59.8% among their urban counterparts [14]. These findings were further supported by evidence showing a decline in COVID-19 vaccination uptake later in the first year relative to the initial rollout period [15]. A study conducted in Wisconsin further highlighted these disparities, finding that individuals living on farms were significantly less likely to initiate COVID-19 vaccination, complete the primary series, or receive a booster compared to non-farm residents within the same region [16].

However, a more nuanced pattern characterized the early phase of vaccine distribution in rural areas. Initially, data suggested higher early uptake among rural residents compared to those in suburban and urban settings [17]. At that time, approximately 40% of surveyed rural populations had received at least one dose of a COVID-19 vaccine, al-though roughly one in five rural residents reported refusing vaccination altogether [16]. By the end of 2022, disparities persisted with approximately one-third of rural adults remaining unvaccinated compared to about 20% of urban adults [18]; similar trends were observed in longitudinal analyses [19]. In Washington state, recordkeeping of COVID-19 vaccination has been formally available since 2022. Initially, 26.9% of the population six months and older in the state received a COVID-19 vaccination; this has decreased to around 20% statewide in subsequent years (19.8% in 2023–24 and 18.5% in 2024–25) [20]. Analysis into vaccination trends in the rural areas of the state describe a different scenario, where early vaccination records denote a 10–15% vaccination rate in 2022–23 followed by a drop to 3–10% COVID-19 vaccination rate in the subsequent two years. For example, in one of the rural counties in eastern Washington, in a county-wide population of about 21,000, in 2023–24 only 777 residents aged six months and older were vaccinated against COVID-19, and the following year, only 828 were vaccinated for a 3.7% and 3.9% vaccination rate, respectively [20].

A growing body of research has identified multiple barriers to COVID-19 vaccination in rural populations [14,15,17,21]. These include lower levels of educational attainment, political factors, residence in mining- or farming-dependent counties, limited access to healthcare services, transportation challenges, and higher levels of poverty [14,15,17,21]. In contrast, less research has focused on successful vaccination strategies in rural settings. Existing studies have largely emphasized public health partnerships [22,23,24]. Evidence suggests that improving vaccination rates in these communities depends on reducing access barriers and fostering strategic collaborations with trusted community members and local healthcare providers [22,23,24], but limited information on “what works” in rural areas is available. This study attempts to add to the available literature on this topic, further identifying successful strategies that have increased COVID-19 vaccine uptake in rural communities. The objectives of this research are to gather experiences of Extension staff in rural communities of Washington State and to identify facilitators of COVID-19 vaccination.

## 2. Materials and Methods

### 2.1. Theoretical Framework

The Consolidated Framework for Implementation Research (CFIR) is one of the most frequently cited frameworks in implementation science and has been widely applied in healthcare research [25,26,27,28]. Since it was introduced in 2009, CFIR has been cited more than 10,000 times [27]. CFIR has often been used in qualitative or mixed-methods studies after the implementation of an intervention to evaluate findings [29]. In healthcare settings, CFIR is particularly useful for identifying barriers and facilitators that influence the successful adoption, integration, and sustainability of evidence-based interventions. CFIR has recently been used to evaluate strategies for vaccinations to prevent dengue [30], malaria [31], human papillomavirus [32], and polio [33]. CFIR is composed of five domains: innovation, outer setting, inner setting, individuals, and implementation process [26]. Individual domains of the CFIR can be utilized to evaluate specific aspects of implementation, and all five domains are often not applicable to one study [29]. The outer setting domain of CFIR was chosen as the theoretical framework for this study because it is designed to evaluate external influences that impact the success of an intervention. In this case, the intervention was offering the COVID-19 vaccine to the public and the external influences were any factors related to acceptance of the vaccine by people living in rural communities. The Outer Setting domain includes seven constructs, including critical incidents, local attitudes, local conditions, partnerships and connections, policies and laws, financing, and external pressure. All seven constructs were used for this study.

### 2.2. Study Design

This study utilized a qualitative descriptive design. Semi-structured key informant interviews [34,35] were conducted by researchers to gather opinions from university Extension employees regarding factors that impacted uptake of the COVID-19 vaccinations during the pandemic. Key informant interviews are in-depth conversations where experts provide detailed insight about a topic [35]. A semi-structured key-informant script (Table 1) was developed using a combination of open-ended and close-ended questions to gather demographic data. Open-ended questions were intended to gather detailed information about the participants’ experiences during the COVID-19 pandemic on the topics of vaccine education (five questions), vaccine outreach (six questions), and barriers and facilitators of COVID-19 vaccination (seven questions). The semi-structured format was chosen because it uses an interview script to guide the conversation but also allows the interviewer to ask additional questions when interesting topics arise organically in the conversation. The interview script was piloted by two student interviewers. Minor adjustments were made to refine the script language and flow. Study methods were reviewed by the Washington State University (WSU) Human Research Protection Program and were found to satisfy the criteria for Exempt Research (Institutional Review Board #20129-001). The Consolidated Criteria for Reporting Qualitative Studies (COREQ) tool was also used to guide for the study design [36].

Artificial intelligence-assisted tools, including Microsoft 365 Copilot (version 365, Microsoft Corporation, Redmond, WA, USA) were used to support interpretation of the quotes to assist with sorting interview data into domains as well as language editing and manuscript organization during preparation of this manuscript. All content was reviewed and revised by the authors, who take full responsibility for the accuracy, interpretation, and conclusions presented.

### 2.3. Study Participants

Washington State University Extension staff were recruited as potential participants for this study. The University Extension program began as part of a federal act in 1862 which set aside federal land to create at least one public educational institution in each U.S. state [37]. Land grant universities were established to provide fair education for all [38]. Extension began as an educational initiative and shifted over time to reflect differing needs in U.S. states, including outreach to rural communities to facilitate leadership training, nutrition skills, and youth mentorship [38]. Extension services are provided in or near each county in the U.S. [39,40].

Washington State University Extension offices have locations in each county in Washington State with an additional location on the Colville Nation tribal land [41]. Extension staff were selected as key informants for this rural health research because they possess a unique awareness of the communities they serve and are representative of every county in the state. Extension staff interface with community members through their role as liaisons between the university and community. Extension staff work, and often also live, in rural communities. They are integrated into the counties they serve with the goal of “creating and delivering targeted research-based knowledge and education” [41]. This familiarity with the community also increases awareness of local challenges and opportunities and the specific demographics, needs, and engagement of the population. Each Extension office serves the rural population within each county in the state, managing the unique culture, demographics, opportunities, and limitations. During the COVID-19 pandemic years, Extension expanded their outreach with their rural communities, providing food and nutrition resources and coordination of medical and community health resources, including vaccination options.

To recruit potential participants, each of the 40 Extension offices was contacted by the primary investigator (K.M.) by email and invited to participate. The recruitment email contained consent and confidentiality information. Email recipients were asked to reply if they were willing to participate in the interview or to suggest someone else at the office if they were unavailable or not interested. Interviews were scheduled via Zoom for willing participants at a time that was convenient for them. A reminder email was sent one week later to those who had not responded, but no further contact was made if there was no reply to the second email. Participants were also offered a $10 gift card in appreciation for their time.

### 2.4. Data Collection

After consenting participants, key informant interviews were conducted via Zoom by two members of the research team. The interviews were conducted by two student pharmacists (GM, SZ) who had been trained in qualitative research methods by an experienced faculty member (KM). A faculty researcher was also present in the Zoom meeting to answer study-related questions, ensure technology was working properly, and support the study, but the faculty member intentionally had minimal participation in the conversation. The researchers chose to have students conduct interviews with the goal of making the participants more comfortable sharing information. One student (GM) was a fourth-year PharmD student and the other (SZ) was in her third year.

Prior to initiating the interview questions, participants were read an IRB-approved informed consent statement and received their verbal consent. Participants were informed the interview would take 20–30 min to complete, and they could end the interview at any time or decline to answer any of the individual questions. They were also informed that any identifying information such as the name of the staff member or location of the Extension office would be redacted from the transcripts by the primary investigator (KM) before sharing the transcripts among the research team and beginning data analysis. The researchers conducting the interview had not met or had a relationship with the participant. In two instances, one faculty researcher (K.M.) had previously met the Extension staff member who was to be interviewed, so the other faculty researcher (M.U.) attended instead. Anonymizing participant responses and ensuring there were no prior connections between the participants and interviewers was done by design to encourage participants to feel comfortable expressing opinions and describing experiences without concerns about giving offense or potential repercussions. Interviews were transcribed using the Zoom transcription feature. Transcriptions and corresponding audio files were reviewed by KM to remove identifiers, check for transcript quality, and correct transcription errors where needed.

Interviews were conducted iteratively and continued until data saturation, defined as the point at which no new information is expected to emerge from subsequent interviews, was reached [42]. Saturation is used in qualitative research in place of statistical power analyses to determine sample adequacy [43]. Upon review of the 21 transcripts, the researchers concluded that saturation had been reached and that additional interviews were unlikely to yield new insights.

### 2.5. Data Analysis

De-identified transcripts were shared among the research team for thematic coding. Deductive qualitative coding methods were used to organize the results into the constructs of the CFIR Outer Setting domain [26]. The researchers met to conduct first-level coding, a systematic process of identifying relevant and repeated concepts to form codes, on one transcript together and then coded the remaining transcripts independently to reduce bias [42]. To enhance analytical rigor, coding decisions were made collaboratively rather than through independent double-coding alone. The initial joint coding of one transcript established a shared understanding of code definitions and application before the remaining transcripts were coded independently.

After first-level coding was completed, the researchers met to perform second-level coding together [42]. Second-level coding is a process of combining codes into themes using the constructs of the theoretical framework [42]. During second-level coding, all researchers reviewed the independently applied codes together, and any discrepancies in code or theme assignment were resolved through group discussion until consensus was reached. This consensus-based approach was used to ensure that thematic assignments reflected a shared, negotiated interpretation of the data across the research team. Notes regarding coding decisions and definitions were maintained throughout the analysis to support transparency and consistency. Quotes illustrating the themes were also gathered and organized. The COREQ guidelines were consulted again as the results were organized and manuscript was drafted to ensure proper reporting [36].

## 3. Results

Responses to email requests to each of the county extension offices resulted in twenty-one participants. These participants represented 34 out of the 40 Extension offices (85%), because several Extension staff reported serving more than one office. These individuals were interviewed during Fall 2023. Extension staff served an average of 1.4 counties (ranged from serving 1 to 3 counties). Details regarding participants’ specific responsibilities were not collected. Participants had been working at WSU Extension for an average of 16.2 years (range 2.5 to 53) and had been at their current site for 13 years (range 1 to 53). Slightly over half of the participants were female (57%). When asked to report the percentage of the area they served that they would classify as rural, the average estimate was 89% (range 25% to 100%). This reflects the demographics of the state, as a very urban county such as King County, where the city of Seattle is located, recognizes 3% of their residents are rural, where the most rural county in the state, Ferry County, is designated as 100% rural [44,45].

**Figure 1:**
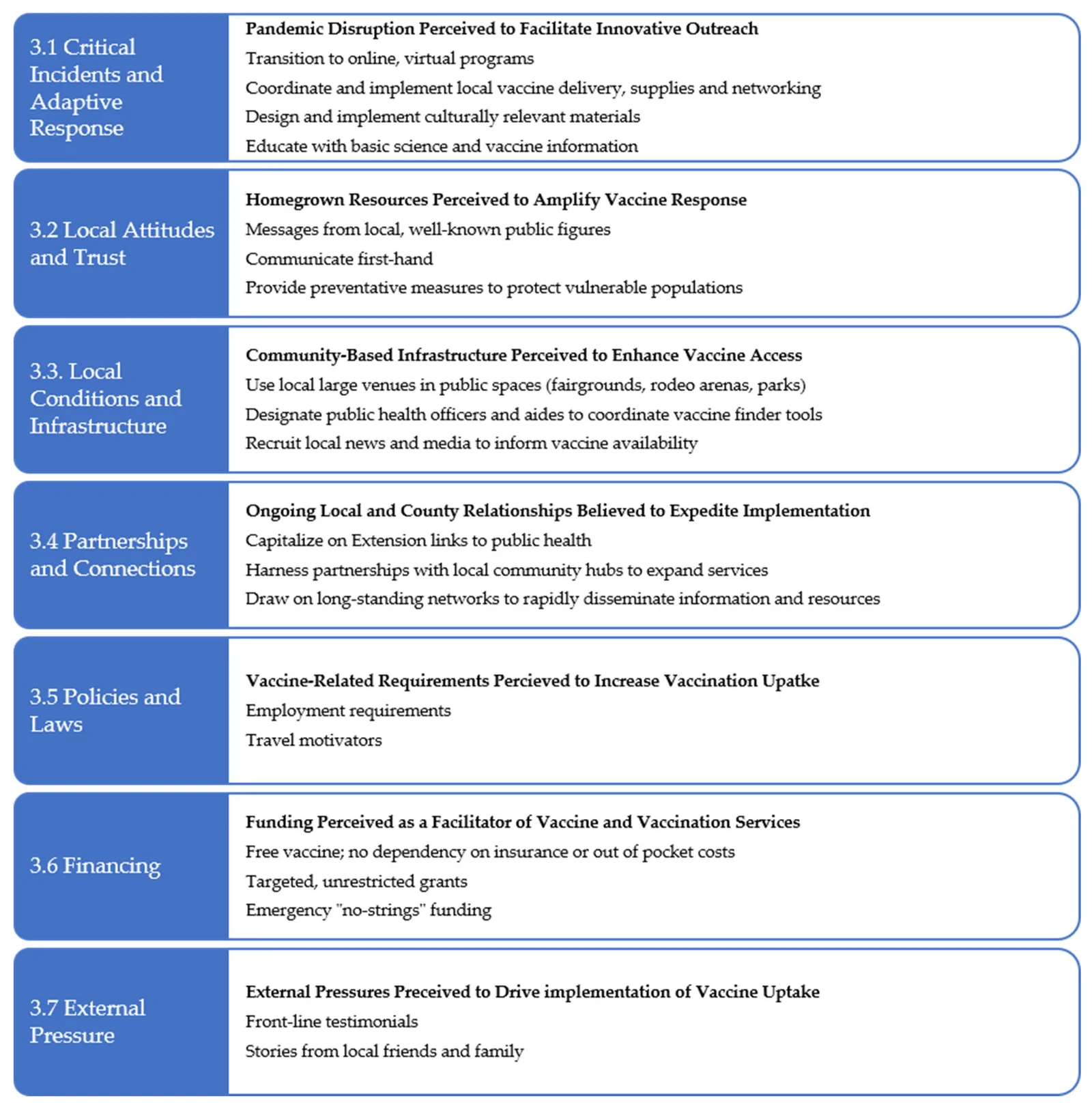
Study results organized within the constructs of the Outer Setting Domain of the CFIR [26]. (See Section 3.1, Section 3.2, Section 3.3, Section 3.4, Section 3.5, Section 3.6 and Section 3.7).

Using the CFIR framework’s Outer Setting domain, multiple factors of COVID-19 vaccination uptake were identified across the seven domain constructs: critical incidents, local attitudes, local conditions, partnerships, policies, financing, and external pressures [26]. Study results organized within the constructs of the CFIR Outer Setting domain are shown in Figure 1.

### 3.1. Critical Incidents and Adaptive Response

In the CFIR framework, critical incidents are defined as the degree to which “large-scale and/or unanticipated events disrupt implementation and/or delivery of the innovation.” [26] The COVID-19 pandemic functioned as a large-scale disruptive event that required rapid adaptation in many ways. Participants described a widespread transition to virtual platforms for education and communication. Participants reported that the shift to virtual learning was challenging and required substantial resources. However, some noted that virtual offerings actually improved reach and engagement because people who lived in remote areas who may not have been able to attend in-person events were able to engage. Several participants reflected on the rapid transition to online programming:

- “We offered online trainings and workshops through our programming, which was actually very successful, and we had better enrollment in some cases. (Participant 10)
- “Being able to meet on Zoom… has been very positive [as] we get to talk to people all over the place.” (Participant 3)
- “All our meetings were online; all our educational events were online.” (Participant 7)

In addition to virtual adaptation, communities implemented creative locally designed solutions that participants believed expanded access to vaccinations and essential services. These included expanding vaccine distribution sites, coordinating delivery of supplies, and leveraging existing networks to fill service gaps.

- “During COVID, 20 more vaccination sites were added around the county [to increase accessibility], so we were working hard with community sponsors to set things up.” (Participant 5)
- “We had people that were taking food and medicine and supplies and delivering them to homes… that service was very well-received.” (Participant 4)
- “I’m on the board for our health clinic… [we asked], can you pick this up in [neighboring county] and bring it over for us? I used every connection I had during that time.” (Participant 16)

Extension staff discussed creating culturally appropriate educational material to improve vaccine understanding among local minority populations. They also emphasized the importance of simplifying complex information so that local people with all levels of education could understand vaccine science.

- “There was very limited information initially for the Spanish-speaking people in our county. They were frightened. They didn’t know what was happening. So we starting including flyers in free lunches to refer them to websites of where to get vaccines in our two counties.” (Participant 2)
- “We hosted a virtual gathering with a highly respected Asian-Pacific Islander woman in the community who got on the TV and radio stations to talk to people about COVID-19 and the vaccine.” (Participant 3)
- “It was getting people involved, as with the Cambodian people, they started asking if we had any information in Cambodian [regarding the COVID vaccine].” (Participant 3)
- “We were focused on creating culturally relevant education around the COVID vaccines for our Hispanic Latino community.” (Participant 19)
- “We have a huge Hispanic agricultural worker population in our county, and the advertising for vaccines was done in both English and Spanish.” (Participant 20)
- “We needed to simplify the science in really digestible format. We turned the vaccine science into a hands-on for people game to understand how our immune system works with the vaccine.” (Participant 18)
- “Even just understanding a little more about the mRNA technology was important… it terrified people that the emergency use was approved so quickly.” (Participant 19)

### 3.2. Local Attitudes and Trust

Damschroder and colleagues define the Local Attitudes construct as the degree to which “sociocultural values and beliefs encourage the Outer Setting to support implementation and/or delivery of the innovation” [26]. Participants consistently reported that individuals in rural communities were more likely to accept the COVID-19 vaccine when information was delivered by trusted local figures, including Extension staff, local healthcare providers, and community leaders. Participants identified trust as a central facilitator of vaccine uptake and Extension was frequently described as a trusted, neutral source of information in the community:

- “Extension workers are trusted community partners.” (Participant 15)
- “People have faith in us. We have a good reputation. We’re science-based.” (Participant 12)
- “If they heard it from a neighbor and someone they trusted, they’ll get vaccinated.” (Participant 17)
- “I think a lot of it goes back to relationships and people you trust. [Residents] trust local individuals a lot more than they do people at the presidential level.” (Participant 8)

Word-of-mouth communication was identified as a particularly influential mechanism for vaccine information dissemination. Personal relationships, including family and close social contacts, played an important role in shaping vaccine decisions. Participants perceived that messaging emphasizing protection of family members and community wellbeing was also effective in motivating local people to get vaccinated. Additionally, empathetic and culturally appropriate communication approaches were described as helping to reduce fear and foster trust.

- “There was the camp that got the vaccine because it’s the right thing to do and they wanted to help their family and society and keep their family and friends safe.” (Participant 18)
- “People will trust their spouse or kid or sister more than they’re going to trust others.” (Participant 19)
- “Getting education out in a way that can be absorbed and is culturally appropriate, literacy appropriate.” (Participant 20, in response to Question 15 regarding outreach activities that were successful.)
- “My father was like super hesitant and I’m not sure he would have got vaccinated if it wasn’t for him having me [Extension staff] to take care of him. I was very patient with answering all of his questions about everything possible [about the mRNA vaccine]. He knew my only motive was to keep him safe, whereas my brother’s strategy would just be to badger him into doing it.” (Participant 19)
- “What a great opportunity to teach social distancing. Don’t touch each other’s fingers when you spread your arms out. That’s your 5 feet distance apart.” (Participant 20, in regard to social distancing instruction to Spanish-speaking farm workers)
- “We partnered with little community non-profits that organized themselves as a hub for folks who are marginalized and to monolingual Spanish-speaking farm workers throughout the area to get access to vaccines and vaccine information.” (Participant 19)

### 3.3. Local Conditions and Infrastructure

Local Conditions are defined as “economic, environmental, political, and or technological conditions” that enable and support implementation [26]. Participants described several structural features of local conditions and infrastructure as facilitators of vaccine update. Thoughtful, community-based vaccination sites were identified in many rural communities that increased access. These included fairgrounds, rodeo arenas, church lawns, and parks. These novel vaccination sites allowed for convenient and flexible access while maintaining social distancing and keeping people outdoors.

- “Utilizing the local fairgrounds helped some with the vaccination process.” (Participant 8)
- “Rodeo grounds… people would queue in line and get poked in the arm as they drove through.” (Participant 13)
- “You could go through and you sat in your car and got vaccinated… then you went on your merry way.” (Participant 4)
- “Public health had a number of stand-up, pop-up clinics at churches and community centers.” (Participant 21)
- “Our health department would drive around and vaccinate you wherever you wanted to get vaccinated.” (Participant 16)

Local public health systems used technology and coordinated information-sharing efforts, such as vaccine clinic finder tools and community outreach platforms, which participants cited with facilitating efficient vaccine roll-out.

- “The vaccine finder website that the state provided… we used that quite a bit.” (Participant 18)
- “The local newspaper was on that. They would do a little write-up each week so that the community could see where to get vaccinated.” (Participant 11)
- “We had an excellent county public health department… working really hard to get quality and timely information out to people. We had a Google Doc that was accessible to key organizations as a master updatable crowd-source list.” (Participant 5)

### 3.4. Partnerships and Connections

The Partnerships and Connections construct of the CFIR described the relationship with “external entities, including referral networks, academic affiliations, and professional organization networks” [26]. Extension staff leveraged pre-existing relationships with local organizations to support education, vaccination outreach events, and emergency services.

- “Supporting the efforts that were ongoing from both Department of Health at the state level, but then also our local health jurisdiction.” (Participant 15)
- “Our county public health officer… we had an excellent county public health department…” (Participant 5)
- “Our office… helped coordinate as far as a location once the vaccine became available… utilizing the local fairgrounds.” (Participant 8)
- “We referred to local clinics [for vaccination distribution] that staged clinics by the local health jurisdiction and set up in parking lots.” (Participant 15)
- “One of the good things the university did was give us freedom… to serve your community the best you can. And for those of us who have been in communities and understand what the process is and where the people are, they know I’m approachable and you can talk to me. (Participant 16)
- “We relied on the vaccine finder website that the state provided and also referred to the local health department because they did the massive vaccine clinics.” (Participant 18).

Participants described community collaboration as a critical facilitator. Extension staff played a key role as intermediaries, linking public health agencies with community organizations and healthcare providers.

- “We just partnered with our community… helping connect people to those services.” (Participant 15)
- “I’m on the board for our local health clinic… and we hired the retired police captain and the old health department administrator who would drive around and vaccinate people wherever they wanted to get vaccinated.” (Participant 16)
- “Yes, several little community non-profits were organizing themselves as a hub for folks who are marginalized to get access to vaccines and vaccine information and other resources.” (Participant 19)
- “We would also tell you the local hospital provides vaccinations… your drug stores provide vaccinations.” (Participant 21)
- “We also helped provide resources and education at vaccine events… even providing just children’s activities at vaccine events.” (Participant 19)

Partnerships also extended to local nonprofit organizations, agricultural organizations, and schools, which participants felt expanded vaccine outreach capacity and improve community engagement.

- “Working with… churches or other community groups to be sponsors of these locations.” (Participant 5)
- “The Baptist preacher had me talk with the church members [about COVID-19].” (Participant 3)
- “We partnered with some connections to get resources to monolingual Spanish speaking farm workers throughout the area.” (Participant 19)

Participants reported that leveraging existing Extension networks enabled rapid dissemination of information and resources. Pre-existing relationships were key for coordinating information and sharing educational material.

- “I used every connection I had during that time.” (Participant 16)
- “We were working with our Emergency Management coordinating team to get them regular updates.” (Participant 5)
- “Our Youth Advocates for Health program did a ton… focused on creating education around the vaccines.” (Participant 19)

### 3.5. Policies and Laws

The CFIR evaluates the extent to which, “legislation, regulations, professional group guidelines and recommendations, or accreditation standards support implementation and/or delivery of the innovation” [26]. Extension staff identified that policies leading to employment- or travel-restrictions for people who were unvaccinated had mixed results. For some, workplace and community group safety requirements during the pandemic were also reported by participants as facilitators of vaccine uptake in rural communities.

- “People got more serious… when they realized they couldn’t go back to work unless the breadwinner was vaccinated.” (Participant 16)
- “When you couldn’t cross borders or get on an airplane without a COVID shot… then people started showing up…” (Participant 15)

Other participants reported that vaccine mandates were seen by people in rural communities as very negative, resulting in people quitting jobs or leaving community organizations.

- “They would drop out of the program… but they were not getting vaccinated… nobody was gonna tell them what to do.” (Participant 2)
- “It got to the point when it was time to get boosters, people said ‘don’t tell your sister [or other family member] because the sister was very much not supporting vaccination. So we go an interesting point in families where we had to keep it a secret so people continued getting their vaccines.” (Participant 19)
- “There was a strong feeling of independence and just not being interested in being told by the government what they should do. It was very much—don’t tread on me. The government can’t advise me and I don’t trust the government.” (Participant 21)

### 3.6. Financing

The CFIR described financing as “the extent to which funding from external entities (e.g., grants, reimbursement) is available to implement and/or deliver the innovation.” [26] Participants consistently identified financial conditions, particularly the availability of free vaccination services, as a major contributor to vaccine uptake. Participants reported that the availability of free vaccines removed financial barriers and increased accessibility in their communities.

- “Cost wasn’t an issue, because when the vaccinations were happening, they were free.” (Participant 20)
- “Making the vaccine available free of charge… I definitely think that was a big player.” (Participant 14)

Funding mechanisms such as grants and emergency funding from governmental organizations supported targeted educational efforts. Receiving this funding also allowed communities to respond quickly as community needs evolved during the pandemic.

- “One of the good things the university did was give us freedom to use the grant funds to serve your community as best you could.” (Participant 16)
- “The funder just said, never mind, just here it is, and use it as fast as you can.” (Participant 19)

Participants also noted that regulations related to requirements for personal protective equipment (PPE) led to opportunities for Extension to engage with community members at distribution sites.

- “We serve as a distribution hub for… masks… and hand sanitizer… hundreds of bottles…” (Participant 19)
- “We were able to provide masks and distribute to some of our livestock producers… and then they could… hand that to their employees.” (Participant 4)
- “One of the updates was now all ag workers… had to supply their employees with masks and hand sanitizer.” (Participant 5)

### 3.7. External Pressures

External pressures, as described in the CFIR “drive implementation and/or delivery of the innovation” [26]. External factors influenced COVID-19 vaccine uptake in rural communities in complex ways. Extension staff reported that the most profound impact on community COVID-19 vaccination rates came from the testimonials of local front-line worker. Participants reported that hearing direct accounts of illness and death from local healthcare providers served as a facilitator in rural communities.

- “I think it’s the front-line workers in COVID… the docs, especially nurses… who saw the real impact of COVID and watched community members die… their testimonial… that this is real… those were the most impactful.” (Participant 13)
- “Unless it impacted your family directly… I think some folks really didn’t believe that people were dying.” (Participant 20)
- “We have a woman who is a highly respected person in the [Asian/Pacific Islander] community. And they got her on TV and all the stations to talk to the people.” (Participant 3)

Conversely, misinformation and political polarization also shaped community perceptions of vaccines negatively. Targeted communication strategies were needed to address these issues.

- “There were conspiracy theories… it was going to give everybody autism… all that was prevalent here just as many other places in the country.” (Participant 7)
- “It went the conspiracy theory… you know they’re tracking you… all that type of stuff… it just wasn’t presented in a logical format…” (Participant 18)

## 4. Discussion

This study gathered the experience of university Extension staff and identified factors that served as facilitators of COVID-19 vaccination uptake in rural communities in Washington State. Since a substantial proportion of rural U.S. residents live in MUAs or belong to MUPs, these findings provide important insight into strategies that may help reduce persistent disparities in preventive healthcare access [7,8]. While national data indicate that a substantial share of rural U.S. residents live in MUA/P-designated areas, the present study did not formally assess the MUA/P status of participants’ specific service areas; MUA/P is referenced here as relevant background context rather than a confirmed characteristic of the study sample. Since inception, Extension has served as a conduit for academic institutions to provide educational outreach to rural and underserved communities [37,38,39,40,41]. Previous research shows that Extension offices are well-positioned to provide direct education, programming, and community outreach, even amid challenging conditions, like those seen during COVID-19 [46].

Extension systems functioned as critical intermediaries between public health systems and rural communities. Given that rural areas nationally are disproportionately designated as MUA/P, where healthcare infrastructure and provider availability are limited [10,11], these intermediary functions may be especially relevant in such contexts, though the specific service areas represented in this study were not formally assessed against MUA/P criteria. Participants described Extension as a trusted, science-based, and locally embedded resource, aligning with its historical role in delivering community-based education [37,38,39,40,41]. The ability of Extension personnel to leverage longstanding relationships and local knowledge was especially valuable in underserved communities, where distrust of external authorities and structural barriers often impede healthcare engagement. These findings align with prior research demonstrating that trusted community organizations strongly influence health behaviors among underserved populations, including Hispanic and Marshallese communities [47], and that dialogue-based interventions can improve vaccine acceptance [48].

Trust emerged as the most salient facilitator reported by participants across all CFIR constructs. Participants emphasized that vaccine acceptance was strongly influenced by interpersonal relationships and local social networks, with rural residents more likely to act on information provided by familiar and trusted individuals than by distant or institutional sources. This finding is consistent with prior studies that emphasized the importance of culturally appropriate, community-based messaging in addressing vaccine hesitancy [22,23,24]. In areas formally designated as MUA/P, where historical inequities and limited access to care may contribute to skepticism, trust-building may be particularly beneficial for effective public health implementation efforts.

Participants described that partnerships and community connections enhanced vaccine outreach and accessibility. Extension staff frequently collaborated with local health departments, schools, faith-based organizations, and agricultural groups to extend their reach. Similarly, previous research has identified partnerships as particularly impactful in MUA/P-designated areas, where resource constraints necessitate coordinated, multi-sector responses [22,23,24]. Leveraging these networks enabled efficient dissemination of information, improved coordination of services, and increased community engagement. Consistent with prior work, Extension’s role as a connector and facilitator strengthened local response capacity [49].

Participants reported that structural and logistical facilitators also played a significant role in improving vaccine uptake. Extension staff believed that the availability of free vaccines reduced financial barriers. This aligns with broader evidence that removing cost barriers is especially important in communities formally designated as MUA/P, where poverty rates are higher and insurance coverage may be limited [6,7,8]. Similarly, participants believed the establishment of flexible, community-based vaccination sites, including mobile clinics and nontraditional venues such as fairgrounds and churches helped address geographic and transportation barriers common in rural settings [14,15,21]. These adaptations highlight the importance of tailoring service delivery models to local conditions in underserved areas.

External influences, including firsthand accounts from healthcare workers, workplace requirements, and travel restrictions, were perceived by participants as factors that shaped vaccine behaviors. While some policy measures increased uptake, they also elicited resistance among certain rural populations, consistent with prior findings that perceived loss of autonomy can act as a barrier to vaccination [21]. Additionally, misinformation and political polarization emerged as persistent challenges, as documented in previous research on rural vaccine hesitancy [14,15,17,21]. These findings reinforce the need for trusted messengers and coordinated communication strategies to counter misinformation.

Overall, these findings suggest that effective vaccination strategies in rural and medically underserved communities require a multifaceted approach that prioritizes trust, leverages local partnerships, and addresses structural barriers to access. These findings suggest that public health efforts targeting MUA/P populations more broadly should incorporate locally trusted institutions such as Extension, invest in community-based infrastructure, and ensure that messaging is culturally and contextually appropriate. These strategies may not only improve vaccination rates but also strengthen community resilience and preparedness for future public health emergencies.

There are limitations to this work that should be considered when interpreting study findings. This study asked Extension staff to participate in key informant interviews, and this approach may have led to selection bias. First, staff who had strong, negative feelings or had experienced trauma during the COVID-19 pandemic may have been less likely to participate. Additionally, the researchers were faculty at the same university where the extension staff were employed. Although results were disseminated anonymously, Extension staff may still have been hesitant to share negative feelings about the work of the university where they are still employed. If selection bias did exist, these factors may have skewed results towards positive findings. This research also has the potential for recall bias since Extension staff were asked to describe experiences more than two years after they occurred. Extension staff may have been inclined to portray their vaccine knowledge or role in vaccine-related activities more favorably because vaccination was a highly visible and socially sensitive public health topic during the COVID-19 pandemic. As a result, the findings may overrepresent positive attitudes toward vaccination and vaccine promotion. Some participants served mixed rural–urban areas and not every reported experience can necessarily be attributed exclusively to rural communities. Lastly, these results represent the opinions of staff in one U.S. state, which may not accurately represent the opinions of their peers in other states. Additional research gathering experiences of people living geographically diverse rural communities may be beneficial.

This study focused exclusively on the CFIR Outer Setting domain, which was appropriate given the study’s aim of identifying external, community-level facilitators of vaccine uptake. However, this focus means the findings do not capture factors from the other four CFIR domains: innovation, inner setting, individuals, and implementation process [26]. For example, organizational-level factors within Extension offices (inner setting), individual Extension staff members’ own attitudes and self-efficacy toward vaccine promotion (individuals domain), or the specific implementation strategies used to roll out vaccination outreach (implementation process) may also have shaped outcomes but were outside the scope of this analysis. Future research applying additional CFIR domains could provide a more complete picture of the multilevel factors influencing rural vaccination uptake. A single-domain approach was chosen to allow for an in-depth exploration of external, community-facing factors, which have been comparatively understudied relative to individual-level determinants of vaccine hesitancy in the existing literature.

The study did not collect quantitative vaccine uptake data. As a result, direct associations between Extension staff’s activities and changes in vaccination rates could not be assessed. However, the findings provide valuable insight into the barriers, facilitators, and strategies influencing vaccine promotion efforts in rural communities. This can lead to informed future interventions and outcomes-focused research.

The COVID-19 response offers several lessons for future public health emergencies in rural communities. Trusted local messengers proved more valuable than any single communication channel, suggesting that emergency preparedness should invest in these relationships year-round. The partnerships that worked best during COVID-19 were also built on pre-existing relationships rather than ones formed under pressure. Future preparedness efforts should establish standing agreements between Extension, public health, and community organizations before an emergency, not during one.

Equitable vaccination access followed directly from removing cost barriers and meeting people where they already gathered: fairgrounds, churches, agricultural events. Shared, regularly updated tools for locating vaccination services, no-cost access, and local delivery are facilitators of a transferable model for future emergencies. Finally, the mobile and pop-up clinic strategies Extension staff described demonstrate that flexible, non-traditional vaccination delivery sites can be adapted to whatever the next public health challenge requires. Local geography and social patterns, rather than fixed clinical infrastructure, should be drivers for decisions regarding where services are offered. These lessons suggest that rural emergency response is strengthened most by sustained, pre-existing investment in local relationships and adaptable infrastructure.

## 5. Conclusions

This study identified trust, community partnerships, and locally adaptive infrastructure as the primary facilitators of COVID-19 vaccination uptake in rural communities as perceived and reported by WSU Extension personnel. Trust was strongest when messages came from familiar, community-embedded messengers, such as Extension staff, local clinicians, and personal networks. Participants reported that partnerships with schools, health departments, and faith-based organizations extended outreach beyond what any single institution could achieve alone. These facilitators offer a foundation for strengthening rural public health preparedness beyond the context of COVID-19 vaccination outreach.

## Figures and Tables

**Table 1:** Semi-structured key informant interview script.

Interview Questions	Topic
How many years have you been an extension agent?	Demographics
2. How many years have you been at your current location?	Demographics
3. Which count(y/ies) in Washington state do you serve?	Demographics
4. Which gender do you most identify with?	Demographics
5. What percent of the area you serve would you describe as rural, urban?	Demographics
6. Where did you get your information to answer questions about the COVID-19 vaccine?	COVID-19 Vaccine Education
7. What training did you receive regarding COVID-19?	COVID-19 Vaccine Education
8. What training did you receive regarding the recombinant mRNA COVID vaccine?	COVID-19 Vaccine Education
9. If people in your community/county asked about where to receive vaccinations, what did your office tell them?	COVID-19 Vaccine Education
10. Regarding the COVID vaccine, what was the most common question you answered from people?	COVID-19 Vaccine Education
11. What were the most common locations where people received the COVID-19 vaccines? (mass clinics)	COVID-19 Vaccine outreach
12. What outreach services (including vaccine services as well as others) did your extension office provide to your communities?	COVID-19 Vaccine outreach
13. What educational outreaches did your office provide during the COVID-19 pandemic?	COVID-19 Vaccine outreach
14. Which other community resources/programs did Extension partner with (such as the health department) to offer outreach services? How was that experience?	COVID-19 Vaccine outreach
15. Which outreach programs were best received regarding COVID-19 information and vaccines? Why do you think they were successful?	COVID-19 Vaccine outreach
16. Which outreach programs were not well received regarding COVID-19 information and vaccines? Why were these not well received?	COVID-19 Vaccine outreach
17. In your community, were there any socioeconomic factors (such as transportation costs, insurance coverage, etc.) that kept people from getting vaccinated?	Barriers and facilitators of COVID-19 vaccination
18. In your community, were there any cultural, ethnic, or religious factors that kept people from getting vaccinated? If yes, please describe.	Barriers and facilitators of COVID-19 vaccination
19. In your community, were there any political factors that kept people from getting vaccinated? If yes, please describe.	Barriers and facilitators of COVID-19 vaccination
20. In your community, were there any education factors that kept people from getting vaccinated? If yes, please describe.	Barriers and facilitators of COVID-19 vaccination
21. What could have been done differently to increase COVID-19 vaccination rates in your area during the pandemic?	Barriers and facilitators of COVID-19 vaccination
22. Were there any factors that increased vaccination interest among the population you serve? If yes, please describe.	Barriers and facilitators of COVID-19 vaccination
23. If there was another public health emergency, do you have any ideas about how to address this better next time?	Barriers and facilitators of COVID-19 vaccination

## Data Availability

The datasets presented in this article are not readily available because the data are part of an ongoing study. Requests to access the datasets should be directed to the corresponding author.

## Abbreviations

The following abbreviations are used in this manuscript:

CFIR	Consolidated Framework for Implementation Research
COREQ	Consolidated Criteria for Reporting Qualitative Studies
COVID-19	Coronavirus Disease 2019
MUA	Medically Underserved Area
MUP	Medically Unreserved Population
MUA/P	Medically Underserved Area/Population
